# Predictive Factors of Efficacy Maintenance after Testosterone Treatment Cessation

**DOI:** 10.3390/jcm8020151

**Published:** 2019-01-29

**Authors:** Min Gu Park, Jeong Kyun Yeo, Sun Gu Park, Woong Na, Du Geon Moon

**Affiliations:** 1 Department of Urology, Men’s Health Clinic, Inje University, Seoul Paik Hospital, Seoul 04551, Korea; uromgpark@gmail.com; 2 Department of Urology, Inje University, Seoul Paik Hospital, Seoul 04551, Korea; yeoluvk@hanmail.net; 3 Department of Clinical Laboratory Science, Daejeon Health Institute of Technology, Daejeon 34504, Korea; sun.park8443@gmail.com; 4 Department of Urology, National Medical Center, Seoul 04564, Korea; woong224@gmail.com; 5 Department of Urology, Korea University, Guro Hospital, Seoul 08308, Korea

**Keywords:** testosterone, testosterone deficiency, testosterone replacement therapy, response maintenance

## Abstract

There is no conclusive evidence as to whether patients with testosterone deficiency (TD) who benefit from testosterone treatment (TRT) must continue the treatment for the rest of their lives. In some patients, the effect of TRT does not maintained after stopping TRT and, some patients show no significant TD symptoms, with normal testosterone levels after TRT cessation. Therefore, we investigated the predictive factors of response maintenance after TRT cessation. A total of 151 men with TD who responded to TRT were followed up for six months after TRT discontinuation. Ninety-two patients (Group I) failed to show response maintenance; 59 patients (Group II) had a maintained response. The groups did not differ in baseline characteristics or the type of TRT (oral, gel, short/long-acting injectables). However, TRT duration was significantly longer (10.7 vs. 5.2 months), and peak total testosterone (TT) level was significantly higher (713.7 vs. 546.1 ng/dL), in Group II than in Group I. More patients regularly exercised in Group II than in Group I (45.8% vs. 9.8%, *p* < 0.001). A multivariate logistic regression analysis revealed that exercise (B = 2.325, odds ratio = 10.231, *p* < 0.001) and TRT duration (B = 0.153, Exp(B) = 1.166, *p* < 0.001) were independent predictive factors of response maintenance. In men with TD who respond to TRT, longer treatment periods can improve the response durability after TRT cessation, regardless of the type of TRT. Additionally, regular exercise can increase the probability of maintaining the response after TRT cessation.

## 1. Introduction

Testosterone deficiency (TD) refers to various hypogonadal symptoms resulting from inadequate testosterone production in males. TD not only causes sexual dysfunctions, such as reduced libido and erectile dysfunction, but also results in physical symptoms, such as fatigue and obesity, and cognitive symptoms, such as depression and poor memory, all of which undermine quality of life. The literature has consistently shown that low testosterone levels are associated with an increased incidence of major adverse cardiac events, including myocardial infarction, stroke, and death [1]. Adequate levels of androgenic steroids such as testosterone and dihydrotestosterone are also very important in the nervous system, which is a major target of androgens that regulate sexual, reproductive, and aggressive behaviors, but also control cognitive abilities, such as language tasks [2,3]. Androgens in the nervous system are involved in development of appropriate neuronal connectivity through the regulation of neurite (dendritic and axonal) outgrowth [2,3]. With the exception of cases undergoing certain infertility treatments, TD can be treated with testosterone gel, oral testosterone, and injectable testosterone [4]. Numerous studies have examined the short-term effects of these testosterone agents. Studies that have examined long-term effects of testosterone agents also show benefits. In follow-up results of the BLAST study, which screened a population of 199 patients for 3.8 years, Hackett et al. reported that long-term testosterone treatment (TRT) was related to a continuous decrease in waist circumference and an increase in erectile function [5]. According to Yassin et al.’s single-center, cumulative, prospective, and registry 10-year study of injectable testosterone undecanoate (TU) treatment on 115 hypogonadal men, endocrine and metabolic parameters continuously improved for up to 7–8 years and were maintained well afterward, without major adverse cardiovascular events [6]. Thus, TRT has positive effects on body composition and insulin sensitivity, as well as on sexual symptoms, which are the most typical TD symptoms [4].

However, there is no conclusive evidence as to whether patients with TD who benefit from TRT must continue the treatment for the rest of their lives. In some patients, the effect of TRT does not persist after treatment cessation, provoking a return to baseline [7,8]. In contrast, some patients show no significant TD symptoms, with normal TT levels, after TRT cessation. Therefore, the purpose of this study was to determine the predictive factors of maintained TRT efficacy after treatment cessation. 

## 2. Experimental Section

From 2011 to 2016, 720 patients with hypogonadism were treated at our institute. Among these, 151 men who responded to TRT and had six months of follow-up after the discontinuation of TRT were included in this retrospective study. Only patients with a serum total testosterone (TT) level of less than 350 ng/dL (measured more than once) and TD symptoms including erectile dysfunction were included. Patients with congenital hypogonadism, such as Klinefelter and Kallmann syndromes, and those with an acquired form of hypogonadism due to ‘organic’ damage to the brain or testis were excluded. During TRT, those who withdrew from the study due to side effects from the drug or lack of treatment efficacy were excluded from the analysis.

TRT includes oral testosterone undecanoate (TU), 2% testosterone gel (T-gel), injectable testosterone enanthate (TE) 250 mg, and injectable TU 1,000 mg, alone or in combination. Oral TU started at 80 mg bidaily, and was adjusted to 40–80 mg bidaily according to the change in TT level. Oral TU was administered after meals. T-gel, in the amount obtained from five pumps of the dispenser, was applied to the inside of both sides of thighs or abdomen early in the morning, and the number of pumps was adjusted to 4–6 times according to the change in TT. TE injection was performed every two weeks, and the interval was adjusted to every two to three weeks according to the change in TT. Injectable TU was injected at 6 weeks after the first injection and then injected once every 12 weeks. The injection interval was adjusted to 10–12 weeks according to the change in TT.

Data were collected from medical records including: TRT duration (duration was the prescription period for oral TU and T-gel, and the period from the last injection plus 3 to 12 weeks was calculated as the treatment period for TE and TU, respectively), type of TRT, age, body mass index (BMI), waist circumference (WC), comorbidities, lifestyle factors (alcohol, smoking, and exercise ≥20 min of moderate intensity three times per week), IIEF (International Index of Erectile Function), AMS (Aging Males’ Symptom) score, and results of baseline serologic tests. Serologic parameters included baseline and post TRT PSA (prostate specific antigen), Hb, Hct, glucose, and baseline total cholesterol, triglyceride, high-density lipoprotein (HDL) cholesterol, and low-density lipoprotein (LDL) cholesterol, baseline serum total testosterone (TT) levels, the highest TT level during TRT, and the most recent TT level at six months after TRT discontinuation. Response maintenance was determined by a normal serum TT level for the most recent TT assessment at six months after TRT discontinuation and a positive response to a global assessment question about TD symptoms. The global assessment question read, “Has the improvement of your TD symptoms resulting from TRT been well maintained since the TRT cessation?”. Blood samples were obtained before 11:00 a.m., and serum TT levels were measured by radioimmunoassay.

The patients were divided into two groups: Group I comprised patients who failed to show a maintained response, and Group II comprised patients who had a maintained response over six months after TRT cessation. Group differences were evaluated using the independent *t*-test or Pearson’s chi-square test, as appropriate, and comparisons between pre- and post-treatment in each group were evaluated using the paired *t*-test. In addition, a multivariate logistic regression analysis was performed to determine the predictive factors of response maintenance. All statistical analyses were performed using SPSS ver. 22.0 (IBM Co., Armonk, NY, USA). For all statistical tests, two-tailed *p*-values lower than 0.05 were considered to be statistically significant.

## 3. Results

### 3.1. Baseline Characteristics

Among 151 patients, 92 patients failed to show response maintenance (Group I; 60.9%) while 59 patients had a maintained response (Group II; 30.1%) over six months after TRT cessation. There were no significant differences between the groups in age, comorbidities, waist circumference, and BMI. There was no significant difference in IIEF and AMS scores between the two groups prior to treatment. Serologic parameters associated with PSA, hemoglobin, hematocrit, and metabolic status did not differ between the two groups. There were also no significant differences between the two groups in the frequency of drinking and smoking. However, the patients in Group II more frequently engaged in regular exercise (>3 times a week for at least 20 min with moderate intensity) than did those in Group I (45.8% vs. 9.8%, *p* < 0.001) (Table 1).

### 3.2. Treatment-Related Factors

**Figure 1:**
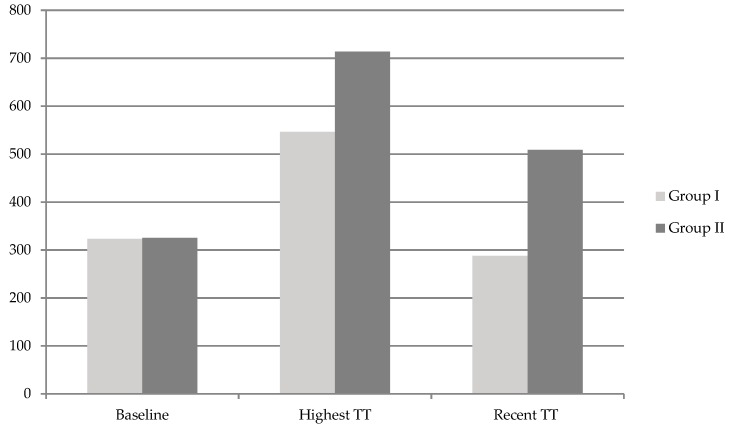
Total testosterone (TT) levels (ng/dL) at baseline, highest TT levels during TRT, and recent TT levels in the six months after TRT cessation in both groups.

Testosterone gel was used in about half of the patients in both groups. In Group II, the proportion of patients using short-acting TE injection was relatively lower than that in Group I, and of the proportion patients using long-acting TU injection was relatively higher than that in Group I. However, there was no significant overall difference in the type of TRT between the two groups. There was no significant difference between the two groups in the mean baseline TT level. However, the peak TT level during TRT was significantly higher in Group II than in Group I (713.7 vs. 546.1 ng/dL, *p* < 0.001), and the increase in TT (highest-baseline) was significantly higher in Group II than in Group I (223.0 vs. 388.5 ng/dL, *p* < 0.001) (Figure 1, Table 2). In addition, the TRT duration was significantly longer in Group II than in Group I (10.7 vs. 5.2 months, *p* < 0.001). After TRT, IIEF, and AMS scores were significantly increased in both groups compared to baseline, but there was no significant difference between the two groups. PSA, Hb, and Hct were significantly elevated after treatment in both groups compared to baseline, but they did not increase beyond the normal range, and there was no significant difference between the two groups (Table 2).

### 3.3. Predictors of Response Maintenance

The multivariate logistic regression analysis revealed that exercise (B = 2.325, odds ratio: Exp(B) = 10.231, *p* < 0.001) and TRT duration (B = 0.153, Exp(B) = 1.166, *p* < 0.001) were independent predictive factors of response maintenance (Table 3).

## 4. Discussion

Various diverse factors are known to affect TT reduction. TD is associated with metabolic conditions, such as diabetes mellitus and obesity, the acute phase disease (a temporary decline, with the exception of factors such as drugs), organic hypothalamic–pituitary–gonadal axis abnormalities, congenital testicular dysfunction, and acquired damage [9,10]. Numerous reports have shown that TT and TD symptoms improve only by controlling these underlying diseases and adopting lifestyle modifications [8,9,11]. However, most of the patients with TD that are seen in clinical practice have accompanying chronic fatigue, decreased physical strength, and depression. Thus, it is often difficult to properly practice lifestyle modifications, which are only effective when strictly carried out for a long period of time. The use of TRT at the beginning of therapy in such patients can lead to an increase in vitality, energy and mood, reducing fatigue and depression, and stimulating the will to live, which can enable patients to more actively pursue the necessary lifestyle modifications [12]. If TRT could be considered as a catalyst for a more effective adoption of lifestyle modifications, it could aid in improving patient compliance, maximizing the therapeutic effect, and maintaining treatment efficacy after TRT cessation. Consistent with this, the results from our previous study showed that practicing a supervised exercise together with TRT was associated with significantly better effects on TT elevation and symptom scores than that with TRT alone [8]. We were able to confirm that the continuation of supervised exercise alone aided in the maintenance of treatment efficacy after TRT cessation [8]. This previous study [8] showed that the group that performed supervised exercise showed greater sustained TRT efficacy than the control group at three months after cessation of TRT, but did not demonstrate that supervised exercise could be a substitute for TRT. A previous other study already reported that the greater the duration of TRT cessation, the more difficult it is to maintain the efficacy of TRT with only life style modifications such as exercise. Although, they did not conduct a supervised exercise program as in our previous study [8], Ng Tang Fui et al. carried out a 56-week double-blind RCT in which 100 patients with obesity and TD participated in a weight loss program of diet and exercise accompanied by TRT. They also observed 64 patients for additional 80 weeks or more while maintaining a weight loss program without TRT [12]. The results showed that body composition improvements that occurred with TRT were not maintained after TRT cessation, but returned to the baseline [12]. However, we believe that life style modification including exercise and diet reduces the risks, cost, and burden of TRT by reducing the TRT duration as much as possible. Furthermore, this approach can improve men’s overall health, as it actively involves lifestyle modifications [12].

Several flawed studies raised concerns regarding the induction of cardiovascular disease (CVD) with TRT [13,14,15]; however, the recent AUA guideline [1] states that “men with low T should be counseled that low T is an independent risk factor for CVD” and subsequent studies [4,16,17,18,19] reported that TRT is relatively safe in terms of the risk for CVD and prostate cancer, as reflected in published guidelines [1,10,20,21,22]. Nevertheless, there are reports that the risk of thromboembolism may increase at the beginning of TRT [23,24]; thus, the safety of TRT remains inconclusive. In terms of prostate safety, the relative contraindications [23,24] for patients with severe lower urinary tract symptoms (LUTS) that were listed in previous guidelines have been excluded from the current guidelines [1,20,21,25,26], as subsequent studies suggested that TRT is helpful for patients with LUTS. Furthermore, the current guidelines have been updated to include statements that TRT can be carefully performed in patients with localized prostate cancer that is well controlled by surgical therapy, if the risk of relapse is not great [1,10,22]. However, in practice, a sudden elevation in PSA level and prostate enlargement have been observed in patients undergoing TRT. In addition, short-term side effects, such as skin issues, edema, flushing, etc., are clearly present in some patients [22]. Therefore, methods to minimize the TRT duration while increasing or maintaining the therapeutic effect, would be helpful for patients.

A previous study reported that symptom improvement was maintained after stopping the treatment with an average of seven months of TRT, even though the TT level returned to its original state [7]. In contrast, another study reported that, in frail elderly men, muscle strength and physical function were not maintained, but rather deteriorated with a decrease of TT, after the cessation of a six-month course of TRT [27]. Additionally, in our previous study, TT, erectile function, and symptoms, returned to pre-treatment levels three months after TRT discontinuation [8]. However, in actual clinical practice, there are patients who improve with TRT and experience maintained TRT efficacy, without deteriorations in symptoms and TT, even if TRT is discontinued. Thus, the present study sought to determine the characteristics of such patients by analyzing data that reflected clinical practice.

The present study revealed a significant difference in peak TT levels between patients with and without maintained efficacy after TRT cessation. In actual clinical practice, the dose and injection intervals for each testosterone agent are adjusted according to the elevated TT concentration level. Since steady-state physiological TT levels are reached after three days of oral TU administration, TT is measured about one week after treatment initiation, and the daily dose is then adjusted to 80–160 mg [28]. Steady-state TT levels are reached after two to three days of the application of 2% testosterone gel, thus, the dose should be adjusted to 2–4 g/day after two to three weeks of administration [28]. Short-acting injectable TE 250 mg is injected once every two to three weeks and the fluctuation in TT is large, resulting in large differences in TT according to the timing of assessment [28]. With long-acting injectable TU, the maximum TT level is reached after 11.4 days and the half-life is approximately 34 days, during which the TT concentration is well maintained within physiological levels [29]. The injection interval must be adjusted to 10–14 weeks, according to the TT level measured at the middle of the interval [29]. As such, there are differences in the maintenance of the TT level, as well as in the frequency of applying or injection, according to each drug. Thus, the peak TT may vary depending on the timing of the blood test. Therefore, while a higher TT level could result in a better maintenance of the treatment effect in the result of present study, it is possible that the observed group differences in the peak TT level and increase in TT relative to the baseline level do not reflect actual clinical implications regarding the effects of the agents on efficacy maintenance.

In the multivariate analysis, regular exercise and treatment duration were identified as factors significantly affecting the durability of treatment. The present study also found that the probability of a maintained efficacy after TRT cessation was 10 times higher in the patients who regularly exercised than in those who did not regularly exercise. Reductions in the serum insulin level [9], oxidative stress [30], the conversion of testosterone to estradiol by aromatase in adipose tissue [31], and a direct increase in the muscular and plasma dihydrotestosterone concentrations resulting from exercise and diet regulation [32] are known to underlie the improvement and maintenance of TT. In the present study, mean BMI was about 25. Therefore, the impact of exercise on loss of visceral fat and insulin resistance might be not relevant or important. Although further studies are needed to determine which exercise is more effective in increasing and maintaining TT, the results of our recent study suggest that the body composition and physical fitness parameters significantly correlated with serum TT are fat-related measures, such as body fat percentage or abdominal fat percentage (rather than muscle mass), and cardio-respiratory fitness, respectively. Therefore, aerobic exercise may be most helpful [33].

In the present study, patients with a maintained therapeutic efficacy had, on average, more than 10 months of sufficient treatment. On the other hand, patients without a maintained therapeutic efficacy had less than six months of treatment, reflecting a relatively short treatment period. In the TRT guidelines, the time until TRT becomes effective varies depending on the symptoms, and symptoms related to sexual function may be improved even after more than one year of treatment [10]. For the patients in the present study, who had the most common symptoms of erectile dysfunction, treatment durations of less than six months were not sufficient to obtain maximum efficacy, and consequently, the patients failed to maintain treatment efficacy after treatment cessation. With the exception of cases in which at least a two-year TRT period is required, such as those with affected bone density, more than one year of sufficient TRT is required, as recommended in the guidelines.

The present study was retrospective in nature and utilized data of patients receiving various agents, which may be considered a disadvantage. However, this study was based on actual clinical treatment patterns, and thus reflects more realistic situations than that in other prospective controlled studies. Because of the differences in the pharmaco-dynamics of each agent, we performed an analysis to determine whether these differences could affect the results regarding the durability of efficacy. The proportion of patients who received TE 250 mg, a short acting-agent, was higher in Group I than in Group II (14.1% (13/92) vs. 8.5% (5/59)). TE 250mg has the disadvantage of causing a large fluctuation in TT, thus the Hct is easily raised when used in older patients and TT is excessively raised to supra-physiologic levels [34]. In addition, Group I had a lower number of patients using long-acting injectable TU compared to that in Group II. The injectable TU has been reported to be superior to other injectable testosterone agents in terms of efficacy, safety, and compliance [35]. Studies that compared injectable TU with other testosterone gels also showed its better efficacy [36,37]. This advantage may have contributed to the maintenance of efficacy in this study. However, we did not find a significant difference in the type of testosterone agent between Groups I and II. It should be noted that about 10% of patients in both groups used two or more agents. The choice of testosterone agent is speculated to be closely related to the cost and convenience of the drug. The drug cost may lead to individual differences in dosage or compliance for oral TU or testosterone gel. While these are disadvantages of retrospective studies, these aspects also should be considered when treating patients with TD.

One of the limitations of this retrospective study is that bone mineral density, body composition, and muscle strength before and after the treatment, which are associated with TD symptoms and TRT efficacy, were not evaluated. In addition, serological results (glucose, total cholesterol, triglyceride, HDL, and LDL) except PSA, Hb, and Hct were not evaluated in all patients before the end of TRT. Since these data were limited, there were limitations in the ability to analyze whether these factors affected maintenance of efficacy.

## 5. Conclusions

In men with hypogonadism who respond to TRT, a longer treatment period can improve the durability of response after TRT cessation, regardless of the type of testosterone treatment. Furthermore, regular exercise can lead to 10-fold increase in the probability of a maintained response after TRT cessation. Thus, the adoption of regular exercise during therapy could reduce the risks, cost, and burden of TRT.

## Figures and Tables

**Table 1:** Baseline characteristics in patients who failed to show a maintained response (Group I) and those with a maintained response (Group II) after TRT cessation.

	Group I	Group II	*p*-Value
N (%)	92/151 (60.9)	59/151 (30.1)	
Age (years)	61.1 ± 9.3	60.4 ± 7.7	0.669
BMI (kg/m^2^)	25.0 ± 2.8	24.9 ± 2.6	0.745
Waist (cm)	86.5 ± 6.8	86.0 ± 6.2	0.640
Comorbidities, N			0.095
None	32/92	19/59	
HTN	28/92	18/59	
DM	18/92	13/59	
Dyslipidemia	8/92	7/59	
Hepatobiliary disease	8/92	4/59	
Pulmonary disease	9/92	5/59	
Chronic kidney disease	8/92	3/59	
Alcohol, N			0.753
	65/92	41/59	
Smoking, N			0.654
	13/92	4/59	
Exercise, N			0.000
Yes	9/92	32/59	
No	83/92	27/59	
IIEF total	27 ± 14.18	29.34 ± 14.1	0.323
Erectile function	11.02 ± 7.03	12.20 ± 7.08	0.316
Orgasmic function	3.71 ± 3.39	4.14 ± 3.35	0.447
Sexual desire	4.23 ± 1.80	4.46 ± 1.87	0.453
Intercourse satisfaction	4.32 ± 3.10	4.68 ± 3.03	0.480
Overall satisfaction	4.05 ± 1.78	4.20 ± 1.81	0.619
AMS total	38.52 ± 9.37	38.86 ± 9.69	0.829
Psycho	9.33 ± 3.63	9.46 ± 3.59	0.827
Somato	14.50 ± 4.27	14.46 ± 4.23	0.952
Sexual	14.70 ± 4.25	14.95 ± 4.32	0.723
PSA (ug/dL)	1.06 ± 0.71	1.07 ± 0.73	0.953
Hb (g/dL)	14.76 ± 1.09	14.82 ± 1.17	0.755
Hct (%)	42.92 ± 3.11	42.70 ± 2.85	0.662
Glucose (mg/dL)	106.86 ± 24.72	105.03 ± 20.54	0.638
Total Cholesterol (mg/dL)	176.12 ± 35.65	181.20 ± 40.24	0.418
TG (mg/dL)	220.01 ± 219.83	233.54 ± 235.79	0.726
HDL (mg/dL)	48.53 ± 10.27	46.96 ± 9.26	0.355
LDL (mg/dL)	114.44 ± 25.93	120.49 ± 30.79	0.206

*p*-value, compared between two groups; BMI, body mass index; HTN, hypertension; DM, diabetes mellitus; IIEF, International Index of Erectile Function; AMS, Aging Males’ Symptoms Score; PSA, prostate-specific antigen; Hb, hemoglobin; Hct, hematocrit; TG, triglyceride; HDL, high-density lipoprotein; LDL, low-density lipoprotein.

**Table 2:** Testosterone treatment-related parameters in patients who failed to show a maintained response (Group I) and those with a maintained response (Group II) after TRT cessation.

	Group I	Group II	*p*-Value
Baseline TT (ng/dL)	323.0 ± 15.4	325.2 ± 19.8	0.221
Highest TT (ng/dL)	546.1 ± 230.3	713.7 ± 139.2	0.000
∆ (Highest–Baseline)TT (ng/dL)	223.0 ± 231.1	388.5 ± 244.0	0.000
TRT duration (months)	5.2 ± 4.4	10.7 ± 9.5	0.000
Type of TRT, N (%)			0.347
Oral TU	15/92 (16.3)	9/59 (15.3)	
T-gel	44/92 (47.8)	31/59 (52.4)	
Injectable TE	13/92 (14.1)	5/59 (8.5)	
Injectable TU	10/92 (10.9)	9/59 (15.3)	
Mixed	10/92 (10.9)	5/59 (8.5)	
IIEF total	35.01 ± 10.04 *	34.92 ± 10.17 *	0.955
Erectile function	14.27 ± 5.34 *	14.14 ± 5.26 *	0.878
Orgasmic function	5.08 ± 2.84 *	5.10 ± 2.80 *	0.957
Sexual desire	5.47 ± 1.84 *	5.30 ± 1.81 *	0.576
Intercourse satisfaction	5.79 ± 2.08 *	5.71 ± 2.16 *	0.817
Overall satisfaction	4.76 ± 1.73 *	4.80 ± 1.76 *	0.902
AMS total	32.332 ± 6.02 *	32.49 ± 6.28 *	0.871
Psycho	7.49 ± 2.11 *	7.56 ± 2.08 *	0.841
Somato	11.42 ± 2.90 *	11.42 ± 2.75 *	0.885
Sexual	13.38 ± 3.80 *	13.47 ± 4.02 *	0.885
PSA (ug/dL)	1.23 ± 0.91 ^¥^	1.31 ± 0.93 ^¥^	0.723
Hb (g/dL)	15.21 ± 1.21 *	15.31 ± 1.05 *	0.755
Hct (%)	45.21 ± 3.81 *	45.70 ± 3.85 *	0.760

*p*-value, compared between two groups using the independent *t*-test; TRT, testosterone replacement therapy; TT, serum total testosterone level; ∆, increase in TT level; TU, testosterone undecanoate; T-gel, 2% testosterone gel; TE, testosterone enanthate; Mixed, in the case of using more than one form of TRT; PSA, prostate-specific antigen; Hb, hemoglobin; Hct, hematocrit; *, *p* < 0.01, compared to baseline in each group using the paired *t*-test; ^¥^, *p* < 0.05, compared to baseline in each group using the paired *t*-test.

**Table 3:** Results of the multivariate logistic regression analysis for the predictive factors of response maintenance after TRT cessation.

	Odds Ratio	*p*-Value
Exercise	10.23	0.000
Periods of TRT	1.166	0.000
Highest TT	1.002	0.192
∆ (Highest–Baseline)TT	1.000	0.978

TRT, testosterone replacement therapy; TT, serum total testosterone; ∆, increase in TT level.
